# Deep transfer learning with fuzzy ensemble approach for the early detection of breast cancer

**DOI:** 10.1186/s12880-024-01267-8

**Published:** 2024-04-08

**Authors:** S. R. Sannasi Chakravarthy, N. Bharanidharan, V. Vinoth Kumar, T. R. Mahesh, Mohammed S. Alqahtani, Suresh Guluwadi

**Affiliations:** 1 Department of Electronics and Communication Engineering, Bannari Amman Institute of Technology, Sathyamangalam, India; 2 School of Computer Science Engineering and Information systems, Vellore Institute of Technology, Vellore, 632014 India; 3 Department of Computer Science and Engineering JAIN (Deemed-to-be University), Bengaluru, 562112 India; 4 Radiological Sciences Department, College of Applied Medical Sciences, King Khalid University, Abha, 61421 Saudi Arabia; 5 Adama Science and Technology University, Adama, 302120 Ethiopia

**Keywords:** Deep learning, Fuzzy ranking, Convolution neural network, Transfer learning

## Abstract

Breast Cancer is a significant global health challenge, particularly affecting women with higher mortality compared with other cancer types. Timely detection of such cancer types is crucial, and recent research, employing deep learning techniques, shows promise in earlier detection. The research focuses on the early detection of such tumors using mammogram images with deep-learning models. The paper utilized four public databases where a similar amount of 986 mammograms each for three classes (normal, benign, malignant) are taken for evaluation. Herein, three deep CNN models such as VGG-11, Inception v3, and ResNet50 are employed as base classifiers. The research adopts an ensemble method where the proposed approach makes use of the modified Gompertz function for building a fuzzy ranking of the base classification models and their decision scores are integrated in an adaptive manner for constructing the final prediction of results. The classification results of the proposed fuzzy ensemble approach outperform transfer learning models and other ensemble approaches such as weighted average and Sugeno integral techniques. The proposed ResNet50 ensemble network using the modified Gompertz function-based fuzzy ranking approach provides a superior classification accuracy of 98.986%.

## Introduction

Being a cancer type provides a higher mortality rate among women, breast cancer is a highly incident cancer type next to lung cancer [1]. The incidence of this cancer type is more found in men rather than women. In the year 2020, over 2.3 million global women remained identified with this cancer type for diagnosis and approximately 6,85,000 have died [2]. Accordingly, somewhere in all parts of the globe, for every fourteen seconds, a woman is getting a diagnosis of this cancer type. This makes this cancer type as the most common type of disease among females in both developed as well as in developing countries. Additionally in the year of 2012, breast cancer was reported in around twelve percent of all newer cancer issues and twenty-five percentage of all cancer types among women [2]. As per the global reports, out of 184 nations, breast cancer is the most recurrently diagnosed illness among women in 140 nations [2]. Due to this, it is the most deadly cancer type among global women. Also, the above fact is true not only for developed nations but also for developing nations.

Another dark side of this problem is that several women do not feel the symptoms of breast cancer at earlier stages [3]. Thus the key to the above problem is earlier identification with timely diagnosis of such cancer. Even though several breast imaging modalities are available, the most effective one for early detection is mammographic imaging. The mammographic procedure can act as a screening tool for the early identification of these cancer types [4]. The imaging procedure has a lower x-ray dose and provides better visualization of breast anatomy i.e., the procedure gives better imaging visual of the microcalcification part of the breast. Based on this, radiologists utilize the obtained mammogram images for precise screening of breast tumors.

Even though there are several advantages found in mammographic procedures and output, there might be a higher risk of getting false positives and false negatives while screening mammogram images by radiologists [4]. In order to overcome this and to assist clinicians, computer-assisted diagnosis (CAD) systems were introduced in 1990 for improving the accuracy of screening mammograms. However, due to the increased demand for early cancer detection, there is always a need for promising CAD tools for breast cancer problems. Thus, the researchers utilize machine learning and deep learning algorithms in an efficient way for solving the abovesaid problem. And by using the deep learning (DL) algorithms in biomedical image classification, there are several solutions obtained and thereby supporting the radiologists and consequently enhancing the accuracy of mammogram screening [4].

Table 1 summarizes some recent works of research used for breast cancer classification. As in Table, Mohammed et al. [5] adopted Logistic, Naïve Bayes (NB), and Decision Tree (DT) algorithms with majority voting ensemble approaches for breast cancer diagnosis. Here, their research attained a classification prediction of 98.1% accuracy with an error rate of 0.01%. Sannasi et al. [6] developed a CAD model based on an extreme learning machine (ELM) optimized using an advanced crow search algorithm for early breast cancer diagnosis. Herein, the research attained a classification prediction of 98.2%, 97.1%, and 98.1% accuracies corresponding to three distinct mammogram databases (DDSM, INbreast, and MI-AS). Muhammad et al. [7] evaluated distinct machine-learning models and ensemble approaches for lung cancer detection. The research paper evaluated the discriminative ability of distinct models such as NB, DT, Support Vector Machines (SVM), and neural network algorithms along with the majority voting and Random Forest (RF) techniques. Herein, the research attained a classification prediction of 90% accuracy using the Gradient-Boosted ensemble Tree approach. Mughal [8] utilized a back-propagation neural network model (BPNN) for breast tumour diagnosis. Herein, the research attained a classification prediction of 98% accuracy for MIAS and DDSM databases. Benzheng et al. [9] developed a CAD approach using CNN models where 97.9% classification accuracy was attained for histopathological images. Naresh and Mishra [10] utilized logistic and neural network algorithms with an ensemble way of classifying breast tumours where they attained an accuracy of 98% results. Mai Bui and Vinh [11] employed a hybrid DL approach using VGG16 and VGG19 architectures with a generative adversarial network for histopathological breast images. Herein, the research attained a classification prediction of 98.1% accuracy. Pratik et al. [12] developed a CAD model using fuzzy concepts with information theory and coalition game. Herein, the research attained a classification prediction of 95% accuracy for a 4-class problem using breast histology images. Khan et al. [13] developed a novel CAD model using CNNs integrated with distinct transfer learning models where their proposed model attained an accuracy of 97.6% for breast cancer diagnosis. Debendra et al. [14] proposed a CAD approach for both ultrasound and mammogram image classification where they attained a maximum of 96.5% accuracy as a final outcome. Moreover, several research works [15–18] employed convolution neural network architectures for solving the breast cancer classification problem. The above discussion reveals that deep learning and ensemble approaches will be used to obtain robust classification frameworks. Also, the breast cancer classification can be further enhanced by leveraging appropriate fuzzy-ensemble methods. In this manner, the proposed approach utilizes transfer learning-based deep learning models and fuzzy-ensemble approaches for breast tumor classification. The motivation behind this and the contributions of the proposed work will be discussed in the next section.

**Table 1:** Some recent works for breast cancer classification

Works	Methods	Outcomes
Mohammed et al. [5]	Logistic, Naïve Bayes (NB), Decision Tree (DT) with Majority voting ensemble approach	Classification prediction: 98.1% accuracy, error rate: 0.01%
Sannasi et al. [6]	ELM Optimized with an advanced crow-search algorithm (Ensemble approach)	Classification prediction: 98.2%, 97.1%, and 98% accuracies for DDSM, INbreast, and MI-AS databases
Muhammad et al. [7]	Various Machine Learning Models with Gradient Boosted Ensemble Approach	Classification prediction: 90% accuracy
Mughal [8]	Back-propagation Neural Model	Classification prediction: 98% accuracy for MIAS and DDSM databases
Benzheng et al. [9]	CNN architectures with a two-class model	Classification prediction: 97.9% accuracy for histopathological images
Naresh and Mishra [10]	Logistic and Neural models with Ensemble approach	Classification prediction: 98% accuracy
Mai Bui and Vinh [11]	Hybrid Deep Learning using VGG16 and VGG19 models	Classification prediction: 98.1% accuracy for histopathological images
Pratik et al. [12]	Fuzzy concepts with information theory andCoalition game	Classification prediction: 95% accuracy for 4-class problem
Khan et al. [13]	CNN with transfer learning approaches	Classification prediction: 97.6% accuracy
Debendra et al. [14]	Deep learning with a 5-learnable layer model	Classification prediction: 96.5% for mammograms and 100% for ultrasound images

## Motivation and contributions

Based on the existing works of literature, fewer scholars employed fuzzy ensemble approaches together with CNN architectures. In general, an ensemble technique is actually a machine-learning approach that combines the predictions of multiple base models into a single final outcome. In specific, the predictions of distinct algorithms are integrated for overall performance enhancement. In specific, this approach is used for fusing the significant properties of its base algorithms, and thereby the overall performance will be enhanced. This results in constituting better results when compared with the outcomes of individual contributing ones. This makes the aforementioned approach robust where the ensembling approach decreases the dispersion or spread of the single model’s predictions. The proposed ensemble approach could be employed as a plug-and-play one by redeeming the weights of the model and applying the same to the test inputs for obtaining final outcomes. This makes the clinicians to employ the proposed approach to be used directly for attaining promising predictions on newer inputs.

As a summary, the proposed work examined the following highlights:

1. The work intends to create a fuzzy ensemble approach that takes digital mammogram image inputs. The research adopted three pretrained transfer learning (TL) CNN networks such as VGG-11, Inception v3, and ResNet-50 for the problem of breast cancer classification.
2. The work employed dense and softmax layers for extricating the feature vectors and for classifying the mammogram inputs with four TL models. An ensemble approach was adopted then for combining the decision scores obtained from the above-mentioned architectures.
3. The research utilized a modified Gompertz function for assigning fuzzy ranks to the individual classifiers based on their decision scores. Herein, fuzzy fusion overtakes the performance of conventional ensemble techniques since the fuzzy ranking-based fusion involves adaptive priority weight assignment for the individual model’s prediction score results.
4. In this approach, the prediction score results rarely become lower as zero. Also, the Gompertz function gets saturated to an asymptote in an exponential manner. This is advantageous for ensembling the individual model’s decision score results since the attained score of a class from a classification model hardly turns out to be truly zero.
5. The research works for the three-class classification and obtained results are assessed using the standard benchmark measures where the proposed framework outperforms the performance of existing models.

**Fig. 1:**
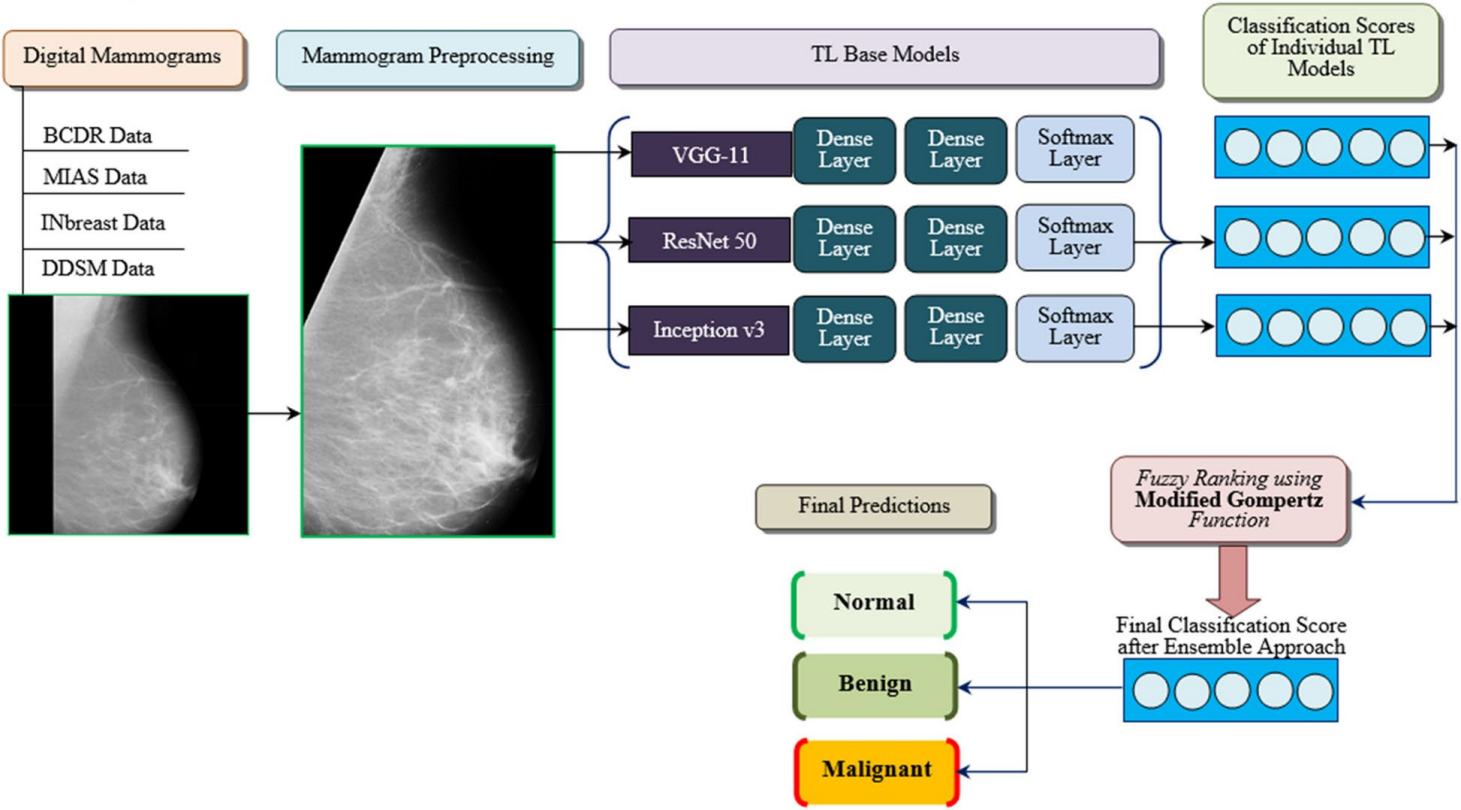
Workflow of the proposed research for breast cancer classification

The workflow of the abovementioned steps of the proposed framework is illustrated in Fig. 1. The appropriate choice of databases and classification models aimed to ensure comprehensive coverage and robustness in the experimentation of our proposed work. At first, BCDR, MIAS, INbreast, and CBIS-DDSM databases are chosen for evaluation. This is because of their wider availability and utilization in the research of breast cancer tumors. The above databases provide a diverse range of mammogram images that represent different aspects of breast cancer pathology and imaging characteristics. Thus, the work aimed to enhance the generalizability of our findings by utilizing multiple mammogram data. This ensures that our proposed methodology performs well across distinct populations and imaging setups. Afterward, as in Fig. 1, VGG-11, Inception v3, and ResNet50 architectures are chosen as base classifiers for our experiments. This is due to their well-established performance in image classification tasks as mentioned in the previous background study and Table 1. That is, the aforementioned transfer learning (TL) models have been widely adopted in the literature and have demonstrated state-of-the-art results across different image databases. Thus, by utilizing these pre-trained deep architectures, the work intended to harness their powerful feature extrication capabilities and at the same time, minimize the need for extensive training on the specific datasets.

In addition, an ensemble way of classification, combined with the modified Gompertz function for fuzzy ranking is proposed to enhance the robustness and accuracy of breast tumor classification outcomes. As from the background study and summary of Table 1, the ensemble methods have been shown to effectively combine the strengths of various base classifiers. And this leads to improved performance over individual models. Herein, the modified Gompertz function allowed us to adaptively integrate the decision scores of the base classifiers. In this way, the ensemble’s predictive capability is optimized. As a summary, the database and classifier selection in the work is driven by the motivation for achieving comprehensive coverage, robust performance, and generalizability in the experimental setup. This will be discussed in detail in the next section.

## Materials and methods

### Mammogram datasets

**Fig. 2:**
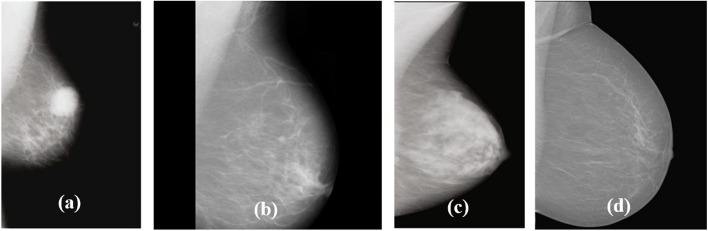
A sample of representative mammograms from four public datasets: (**a**) BCDR (**b**) MIAS (**c**) CBIS-DDSM (**d**) INbreast

The evaluation of the proposed research as shown in Fig. 1 is carried out using multimodal datasets for breast cancer classification. Herein, four distinct mammogram databases that are publicly accessible are employed: the Breast Cancer Digital Repository (BCDR) [19], the Mammography Image Analysis Society (MIAS) [20], INbreast [21], and the Curated Breast Imaging Subset of Digital Database for Mammography Screening (CBIS-DDSM) [22] datasets. The research employed an equivalent amount of normal, benign, and malignant mammographic images from the aforementioned datasets. In this way, the research adopts 986 digital mammograms for each class output and so the work focuses on the evaluation of balanced data. By using these three class inputs of each 986 mammograms, the three transfer learning models were trained for discriminating whether an individual had breast cancer or not. Figure 2 illustrates a sample of representative mammograms from four distinct aforementioned public datasets.

### Preprocessing of mammograms

During the mammogram image acquisition, the impulse noise present in the BCDR, MIAS, INbreast, and CBIS-DDSM datasets is removed using the adaptive median (AM) filter [23]. This type of filter removes the noise in an adaptive manner such that the filter does not distort the non-affected pixels. After noise removal, the darker regions on either part of the mammograms are manually cropped for the four datasets. Now, the significant part of mammogram preprocessing is the removal of pectoral muscles for mediolateral oblique view (MLO) images. In all four mammogram datasets, the pectoral muscles are removed by using the following steps,

(i) The pectoral muscle (PM) which is basically a homogenous region consisting of brighter pixel areas, is identified.
(ii) This PM is usually present in either of the upper left or right-side corners of images. So left-side view mammograms are flipped to resemble right-side view images, thereby consistency in the mammogram orientation for analysis is ensured.
(iii) Now the flipped images are divided uniformly into 4 equivalent quadrants; notably, the PM is found at the leftmost-upper quadrant as shown in Fig. 2.
(iv) Subsequently, it is found that the gray-level intensity in the leftmost upper quadrant (PM regions) is quite different from the intensity distribution over other quadrants.
(v) Finally, global thresholding [24] is applied over the mammograms for PM removal.

The above steps help to standardize the intensity levels across the mammogram images and thus more accurate feature extraction by the transfer learning models will be attained.

### Deep transfer learning models

Transfer learning is a methodology that involves the reuse of already trained CNN architectures for solving newer but related problems [25]. This subsection covers the concept of three transfer learning models namely VGG-11, ResNet50, and Inception_v3 architectures. These pre-trained architectures are trained previously on large-scale image datasets (ImageNet) and exhibit exceptional capabilities in capturing intricate features from applied inputs. By utilizing the concept of transfer learning, the already learned representations from these models could be applied effectively to the proposed problem of breast cancer identification, even with limited labeled data. From these models, the features are extracted from multiple layers which facilitates the models to capture both lower-level and higher-level image representations. The obtained features encode essential characteristics of the mammogram inputs, such as shape, texture, spatial, and other patterns. Thus, the extricated features will serve as informative representations of the mammogram data, facilitating subsequent classification using Fuzzy-ranking-based classification.

#### VGG-11 model

**Fig. 3:**
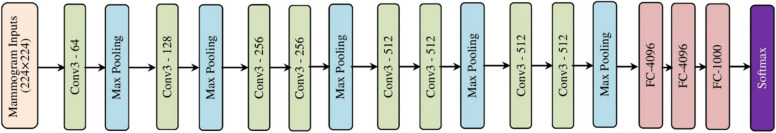
Architectural diagram of VGG-11 model

The Visual Geometry Group is the expansion of the VGG-11 model and it is popular among ML researchers for solving problems in several fields of artificial intelligence [26]. The earlier derivative CNN models of AlexNet [27] intended to work on attaining reduced window size and stride in the first layer of convolution. But the VGG model focuses on another significant improvement for CNN architecture: depth. The model’s convolution layers utilize a smaller receptive field, that is, the model employs the concept of minimizing the convolution filter size to a $3\times3$ kernel. By using this fixed kernel concept, there is a feasibility of adding several weight layers extending up to 19 layers. The employed VGG-11 model comprises eleven layers where eight are convolution layers and the rest are fully connected layers [26]. Here the window size of the pooling layer is $2\times2$ with the size of the stride being considered as two. This is used for minimizing the convoluted feature input size whereas the translational invariance of the model is preserved. A softmax function is used as the final classification layer for the three-class classification problem of breast cancer. Herein, the ReLU function is used as an activation function for all the hidden layers. The detailed architectural diagram of the model is illustrated in Fig. 3.

#### ResNet50 model

**Fig. 4:**
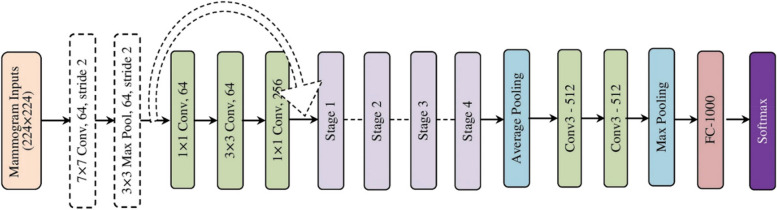
Architectural diagram of ResNet50 model

The detailed architectural diagram of the ResNet50 model is depicted in Fig. 4. As in the figure, the model’s architecture consists of four stages [28]; the max-pooling and initial convolution of the model is carried out through the kernel size of $3\times3$ and $7\times7$. Then, the model has stage 1 where three residual blocks with three layers each are present. Here, the kernel size employed for performing the process of convolution in all the layers of stage 1 is different [28]. The identity connection of the model is represented in the architecture of Fig. 4 through curved arrows. Also, the convolution process carried out in the residual block is done using the stride value of two, so that the mammogram input size would be decreased to half but the width of the channel gets increased by a factor of two. When the input progresses from the first stage to the next, thus the input size and the channel width will become reduced (0.5) and increased (twice). In final, the model consists of an average pooling layer and subsequently, a fully-connected layer with a softmax function is used.

#### Inception v3 model

**Fig. 5:**
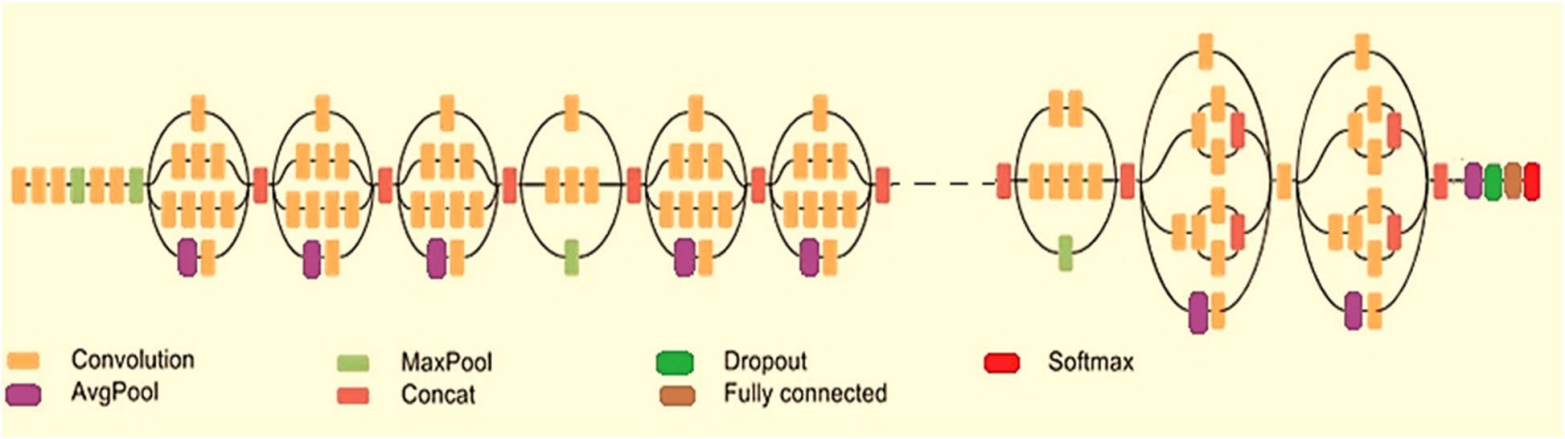
Architectural diagram of inception v3 model

The detailed architectural diagram of the Inception v3 model [29] is depicted in Fig. 5. As in the figure, the model is one of the Inception family of convolution neural architecture that provides greater classification ability using the process of transfer of learned parameters. Additionally, the model is basically an extensive version of the GoogleNet CNN architecture that utilizes batchnorm [30] extensively in the activation layer regions. Once the input images are applied to the network, the images progress through distinct layers of convolution where the deep features are extricated. In this model, the inception blocks are beneficial for computing on distinct filters of feature extrication through concatenation of computation results into a feature map as given in Fig. 5. Moreover, the Inception v3 model reduces the computational complexity through the reduction in the number of parameters.

In summary, VGG-11 provides abstract features learned by the model, such as edges, textures, and object parts. ResNet50 captures complex hierarchical features from the applied mammogram data and provides more discriminative representations when compared with shallower architecture models. The inception v3 model captures multi-scale features and spatial hierarchies within the applied mammogram data and thus provides robust feature representations for the next classification task.

## Proposed framework – fuzzy rank ensemble technique

The ensemble approach considers the significant features of all the base classification models and so provides a substantial improvement in the overall performance. The work considers the popular and traditional fuzzy ensemble functions such as Weighted Average (WA) and Sugeno Integral (SI) for comparing the performance of fuzzy ranking using the modified Gompertz function.

### Weighted average

This approach of predicting the fuzzy WA was initially developed by Dong et al. [31]. And this approach seems to be a straight-forward and simple one since the approach averages the final predictions obtained through distinct weak learners. In addition, the approach makes use of weight assignments to every individual learner for obtaining the final predictions, that is, it will not consider the serial and parallel computation. Consider the fuzzy number sets ${W}_{1},{W}_{2}\dots {W}_{n}$ and ${A}_{1},{A}_{2}\dots {A}_{n}$ which are defined on the corresponding universe sets ${Z}_{1},{Z}_{2}\dots {Z}_{n}$ and ${X}_{1},{X}_{2}\dots {X}_{n}$. In this case, the fuzzy WA $\left(y\right)$ can be defined as when $f$ is denoted as a function that maps from ${Z}_{1}\times {Z}_{2}\times \dots {Z}_{n}\times {X}_{1}\times {X}_{2}\times \dots {X}_{n}$ to the universe of $Y$ is [31]

1 $$y=f\left({x}_{1},{x}_{2},\dots {x}_{n},{w}_{1},{w}_{2},\dots {w}_{n}\right)=\left(\frac{{w}_{1}{x}_{1}+{w}_{2}{x}_{2}+\dots +{w}_{n}{x}_{n}}{{w}_{1}+{w}_{2}+\dots +{w}_{n}}\right)=({w}_{1}{\prime }{x}_{1}+{w}_{2}{\prime }{x}_{2}+\dots +{w}_{n}{\prime }{x}_{n})$$

In the above equation, ${x}_{i}\in {X}_{i}$ and ${w}_{i}\in {Z}_{i}$ for each $i=\text{1,2},\dots n$. Also, $W_i{^\prime}$ in the equation denotes the normalized weight computation.

### Sugeno integral (SI)

The approach of using Sugeno fuzzy integral was developed by Takagi-Sugeno [32] that involves the fuzzy rule generation from a database having both input and output representations. Herein, the input parameters are hazy whereas the outputs are non-hazy. During the implementation of the approach, Takagi Sugeno adopted the weighted average calculation for determining the crisp results and so the approach is computationally effective [33] and might be easily employed together with any adaptive and optimization approaches.

### Fuzzy rank ensemble with modified Gompertz function

As in literary works, the conventional ensemble approaches consider an equivalent priority for the decision scores obtained using distinct classifier types and these classification models use the precomputed weights for the calculation. The primary problem with the above considerations used in the conventional approaches is the creation of static weights that are hard to alter in the final phases where the classification of test inputs is done. But, the prediction scores of every base classification model are considered for each test phase individually in the proposed fuzzy ranking-based ensembling method. In this manner, the classification problem is implemented and attains improved classification results using this ensemble approach. Additionally, the abovesaid approach is a dynamic one so that changing of any weights for distinct test samples is not required.

The proposed work utilized the concept of the Gompertz function that can be mathematically illustrated as: [34]

2 $$f\left(t\right)=m{e}^{{-e}^{n-pt}}$$

**Fig. 6:**
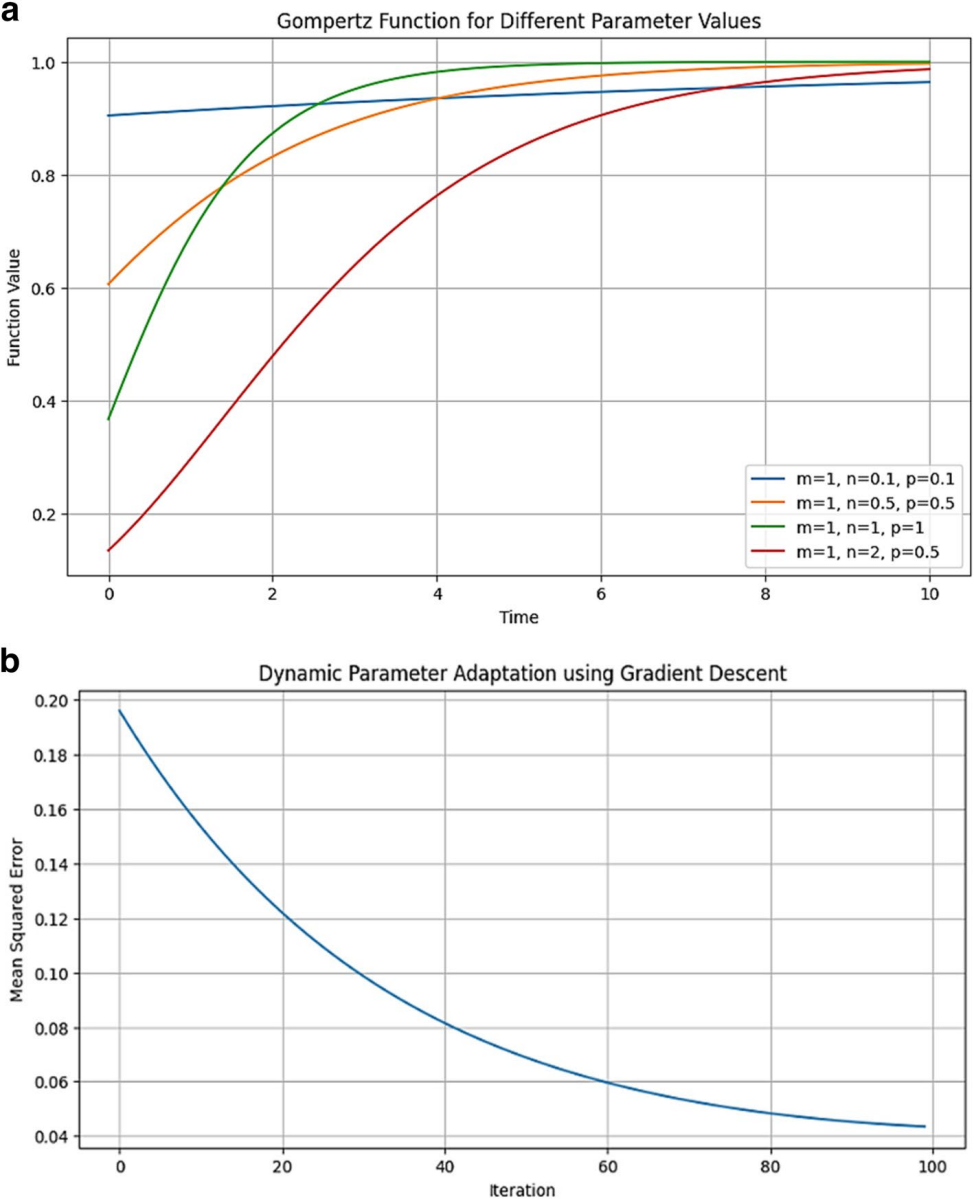
**a** Gompertz function for different parameter values. **b** Modified Gompertz function for the proposed work

In Eq. (2), the asymptote is notated as $m, n$ is used for setting the displacement over the horizontal axis whereas $p$ is for vertical scaling and $e$ denotes the Euler number. Figure 6a illustrates the graph of Gompertz function with changing values of variables $m,\,n,\,and\,p$. These parameters are automatically chosen with respect to the minimum mean square error loss function for the employed TL models. This dynamic parameter adaptation of the modified Gompertz function is attained using gradient descent search algorithm [35] as given in Fig. 6b. Thus, the Gompertz function is modified with dynamic parameter adaptation for the proposed approach. In the proposed research, the modified Gompertz function is employed for the three-class breast cancer problem. Let $N$ be the number of classification models taken into account so that there will be a $N$ number of prediction score values obtained for every mammogram in the test phase of all databases. As illustrated in sub-section 3.3, the work utilizes three deep TL models so that the value of $N=3$. If $L$ is considered to be the number of output classes of employed mammogram databases, then

3 $${\textstyle\sum_{l=1}^L}\;S_l^{\left(n\right)}\;=1;\forall n,n=\text{1,2},3,\dots,N$$

In the above equation, the parameter $S$ denotes the prediction scores of each output label for each taken input image during the generation of fuzzy rankings. The fuzzy ranking of the ${n}^{th}$ classification model for the ${l}^{th}$ output class can be calculated as [36].

4 $$R_l^{\left(n\right)}=(1-\epsilon^{{-\epsilon}^{-2\times S_l^{\left(n\right)}}});\forall n,l$$

Here, the values of $l$ and $n$ range from $\text{1,2},3\dots L$ and $\text{1,2},3\dots N$. The proposed work involves the three-class classification namely normal, benign, and malignant mammogram cases pertained to four different databases. Herein, the work considers the number of top classes as $k=2$. The below Eqs. (5) and (6) are framed for the calculation of fuzzy ranking denoted as $FR{S}_{l}$ and $CCF{S}_{l}$ representing the complementary confidence factoral sum for the considered output label $l$. In this case, let if the class $l$ fails to be in any of the three classes, then a penalty value representing ${P}_{l}^{R}$ and ${P}_{l}^{CF}$ are imposed on to the respective label. Finally, the final prediction of the class label corresponding to the taken mammogram $X$ can be determined using the product of two terms: $FR{S}_{l}$ and $CCF{S}_{l}$. And then calculating the minimum value among all the class labels as illustrated in Eq. (7).

5 $$FRS_l={\textstyle\sum_{i=1}^N}\left\{\begin{array}{c}R_l^{\left(i\right)},ifR_l^{\left(i\right)}\in K^{\left(i\right)}\\P_l^{\left(R\right)},Otherwise\end{array}\right.$$

6 $$CCFS_l=\frac1N{\textstyle\sum_{i=1}^N}\left\{\begin{array}{c}{CF}_l^{\left(i\right)},ifR_l^{\left(i\right)}\in K^{\left(i\right)}\\P_l^{\left(CF\right)},Otherwise\end{array}\right.$$

7 $$class\left(X\right)=\text{min}\left\{{FRS}_{l}\times {CCFS}_{l}\right\} \forall l=\text{1,2}, and\,3.$$

## Experimental results and evaluations

### Experimental environment

The experimentations carried out in the proposed work are done using the Python framework with Google Colab as an IDE. The system used for the implementation has the configuration of 8 GB of RAM with an NVIDIA GPU running on the backend as the Keras framework. The work intends to categorize the mammogram inputs as either normal or benign or malignant cases and the research utilizes similar hyperparameters for the training of three employed TL models (VGG11, ResNet50, and Inception v3). After input preprocessing, the mammograms are resized eventually to $224\times 224$ and applied then to the transfer learning models for feature extrication. Herein, the architecture of the three pretrained TL models is frozen excluding the layers next to the convolution layers. For classification and further evaluation, the inputs are split into a training set with 70% of data inputs and a testing set with 30% of mammogram inputs.

After the extrication of feature vectors from the TL models, the weights of the convolution process are kept constant and further CNN layers such as pooling, fully connected, and dense layers are included based on the respective deep architecture. Moreover, the softmax activation function has been incorporated at the last of all three employed TL models. This layer’s output results in a probability distribution on the predicted class labels and this could be defined as the confident score obtained from the classification models. The work utilized a learning rate of $0.001$ and epochs of 50 for avoiding the overfitting problem in TL models during the training and testing stages of mammogram classification. The optimizer employed was ADAM [37] for gradient descent with the abovesaid learning rates and $0.9$ as $\beta$ value. During the implementation of the proposed framework, the process of training is experimented with using distinct learning rates, epochs, and batch sizes, and these experimented values are taken for the TL models. After feature extrication, the work utilized 2 dense layers having 4096 neurons each at the end having a ReLU function. Finally, the last layer is included with 3 softmax output nodes since the work involves three class classifications as depicted in Fig. 1.

### Results and experimentations

The fuzzy ensemble-based ranking CAD model using the modified Gompertz function assigns the weights adaptively on the decision score of the classification models for providing the final output. The work utilizes the standard performance evaluation measures such as Precision, Recall, and F1 score for the employed three-class classification problem. The above three metrics are calculated for each class: normal, benign, and malignant cases, and then the overall accuracy is calculated for the problem. Afterward, the above-obtained results are then validated through Cohen’s kappa validation $\left(\kappa \right)$ metric [38]. The summary of performance measures is given below.

- The Precision metric provides a measurement of the proportion of correctly predicted positive severity out of all mammogram severities predicted as positive [38]. It is calculated as the ratio of true positives $\left(TP\right)$ to the sum of true positives and false positives $(TP+FP)$.
- Recall, also known as Sensitivity, provides a measurement of the proportion of correctly predicted positive severities out of all actual positive mammogram severities [39]. It is calculated as the ratio of true positives $\left(TP\right)$ to the sum of true positives and false negatives $(TP+FN)$.
- The next one, F1 score is a metric that provides the measurement of harmonic mean of recall and precision. This ensures a balance between the precision and recall metrics [40]. It is calculated as two times the product of precision and recall divided by their sum.
- As a final point, overall accuracy provides the measurement of the proportion of correctly classified cases out of all mammogram cases taken as inputs. This metric provides a general assessment of the classification model’s performance across all mammogram classes [41]. The metric is calculated mathematically by summing the number of correctly classified cases $(TP+TN)$ across all mammogram severity inputs and dividing by the total number of instances $(TP+FN+TN+FP)$.
- Finally, Cohen’s kappa statistic metric is utilized to measure the agreement between the predicted classifications and the true classifications This validation metric provides a measure of inter-rater agreement that considers the possibility of agreement occurring by random chance [39]. Herein, a higher kappa value indicates better agreement between the predicted and true classifications.

The aforementioned performance measures (Precision, Recall, F1 Score) are calculated individually for each class (normal, benign, and malignant) for the robust evaluation of assessing the performance of the classifiers in distinguishing between different mammogram severities. By employing the above evaluation metrics namely precision, recall, F1 score, overall accuracy, and Cohen’s kappa, the effectiveness of the proposed fuzzy ranking ensemble approach is effectively assessed in accurately classifying mammogram inputs into normal, benign, and malignant severities. Thus, the above-discussed performance measures provide insights into the classification model’s performance across different targets and its agreement with the ground truth labels. In this manner, the paper ensures a robust evaluation of the proposed approach.

**Fig. 7:**
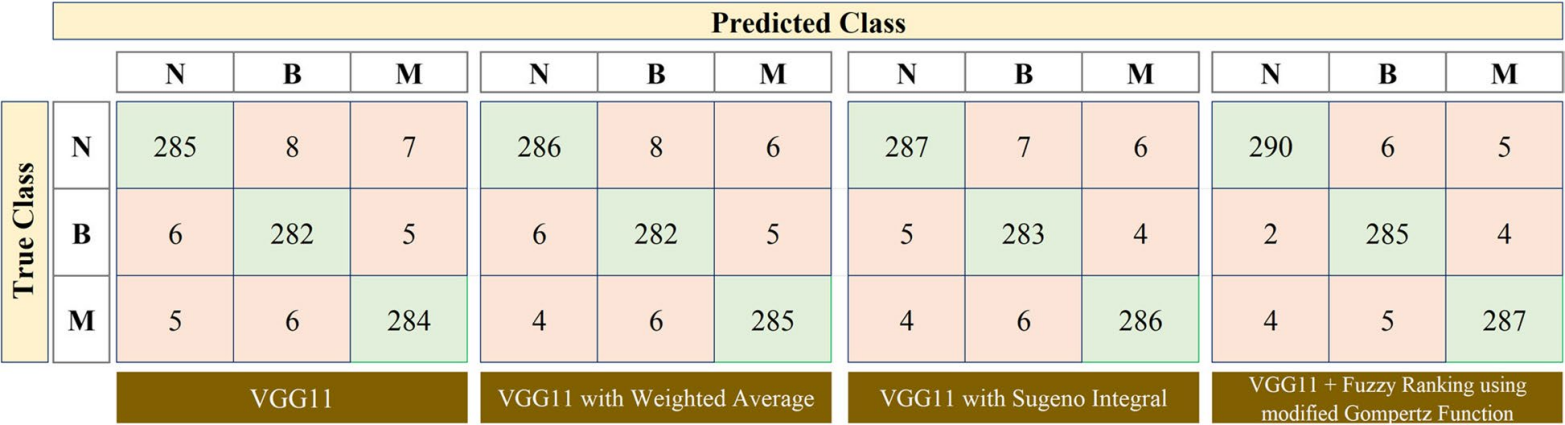
Confusion matrix obtained for VGG11 model and its ensemble approaches (N – Normal, B – Benign, M – Malignant)

**Fig. 8:**
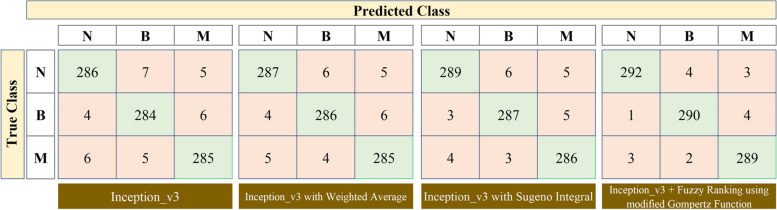
Confusion matrix obtained for Inception v3 model and its ensemble approaches (N – Normal, B – Benign, M – Malignant)

**Fig. 9:**
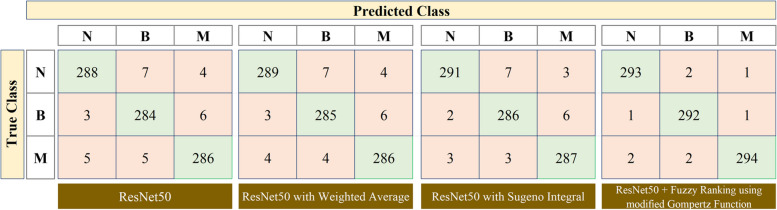
Confusion matrix obtained for ResNet50 model and its ensemble approaches (N – Normal, B – Benign, M – Malignant)

Specifically, by evaluating precision, recall, and F1 score for each class, we can assess the classifier’s ability to correctly classify instances from each class while considering both false positives and false negatives. And all the above metrics are constituted from the elements of the error matrix or confusion matrix. In this way, the experimentation of the research yields the confusion matrices for the three TL models and they are illustrated in Figs. 7, 8 and 9. In these figures, the confusion matrix of the standalone TL model along with their ensemble approaches such as weighted average, Sugeno integral, and the proposed Fuzzy ranking-based ensemble method using modified Gompertz function are depicted.

**Fig. 10:**
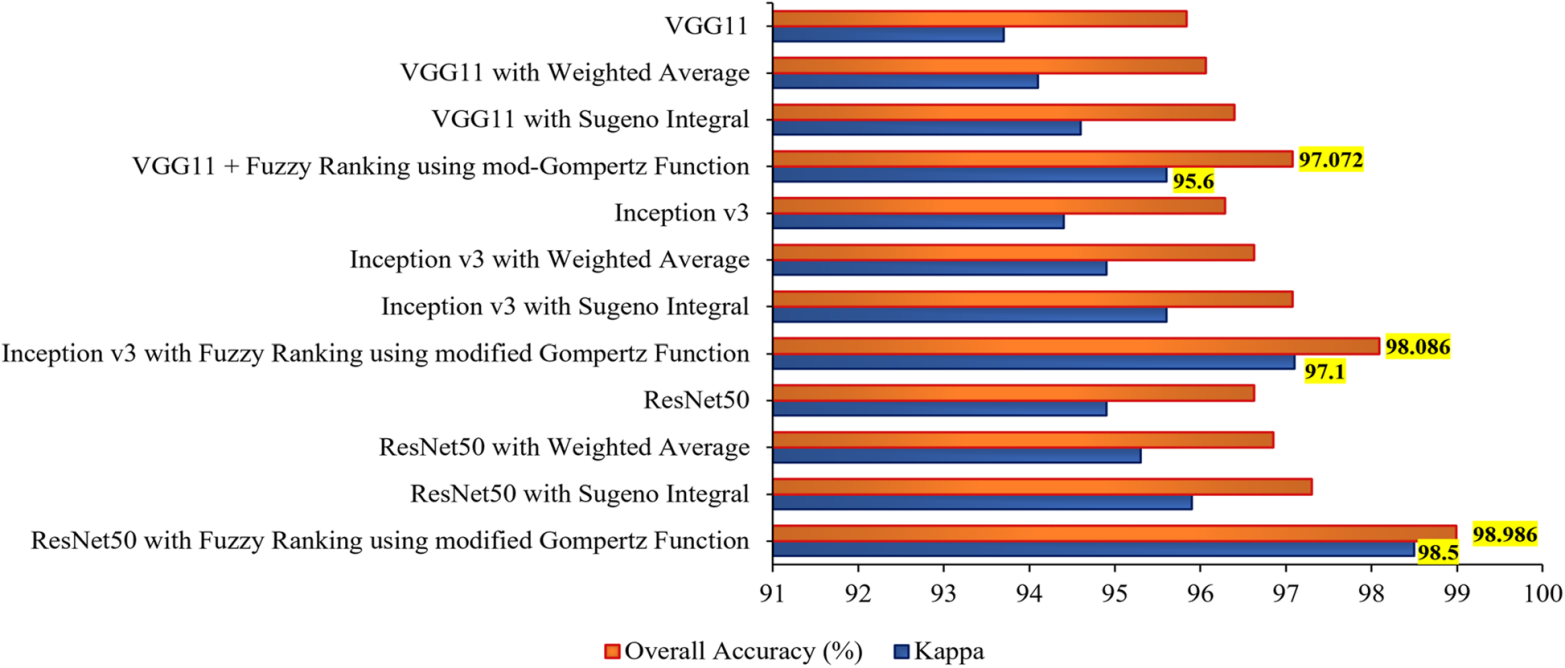
Comparative performance of transfer learning models with the proposed approach of different fuzzy ensemble techniques

Based on the obtained confusion matrix, the results of the VGG11, Inception v3, and ResNet50 transfer learning models are tabulated in Table 2. The results summarized in this table reveal that the proposed Fuzzy ranking ensemble using the modified Gompertz function outperforms other fuzzy approaches for all the three employed transfer learning architectures: VGG11, Inception v3, and ResNet50. Next to the proposed approach, the Sugeno integral function of the fuzzy approach performed well for all the deep learning models. The above results are comparatively presented as a graph in Fig. 10 where the overall accuracy and validated kappa $\left(\kappa \right)$ are taken into account. The values of $\kappa$ are generally calculated in the range of -1 to 0 to + 1 where the values obtained from − 1 to 0 represent that there is a highly disagreement among the classes for the classification models in predicting breast cancer severities. And $\kappa$ values closer towards + 1 represent that there is a good agreement among the classes that exist in discriminating the severities of output classes. However for better comparison analysis, in Fig. 10, the obtained $\kappa$ values are scaled to the range of 0 to 100% as similar to the overall accuracy metric.

**Table 2:** Performance summary of the deep TL models using different ensemble methods applied on the testing set of mammogram inputs (N – Normal, B – Benign, M – Malignant)

TL Models	Output Classes	Precision (%)	Recall (%)	F1 Score (%)	Overall Accuracy (%)
VGG11	N	95.013	96.284	96.149	95.833
	B	96.246	95.27	96.088	
	M	96.271	95.946	96.113	
VGG11 with Weighted Average	N	95.333	96.622	96.225	96.059
	B	96.246	95.27	96.151	
	M	96.611	96.284	96.202	
VGG11 with Sugeno Integral	N	95.667	96.959	96.479	96.396
	B	96.918	95.608	96.487	
	M	96.622	96.622	97.395	
VGG11 with Fuzzy Ranking using modified Gompertz Function	N	96.346	97.973	97.474	97.072
	B	97.938	96.284	97.321	
	M	96.959	96.959	97.386	
Inception v3	N	95.973	96.622	96.224	96.284
	B	96.599	95.946	96.157	
	M	96.284	96.284	96.183	
Inception v3 with Weighted Average	N	96.309	96.959	97.316	96.622
	B	96.622	96.622	97.259	
	M	96.939	96.284	97.212	
Inception v3 with Sugeno Integral	N	96.333	97.635	97.498	97.072
	B	97.288	96.959	97.351	
	M	97.611	96.622	97.333	
Inception v3 with Fuzzy Ranking using modified Gompertz Function	N	97.659	98.649	98.489	98.086
	B	98.305	97.973	98.392	
	M	98.299	97.635	98.244	
ResNet50	N	96.321	97.297	97.516	96.622
	B	96.928	95.946	96.773	
	M	96.622	96.922	97.491	
ResNet50 with Weighted Average	N	96.333	97.635	97.689	96.847
	B	96.939	96.284	97.258	
	M	97.279	96.622	97.614	
ResNet50 with Sugeno Integral	N	96.678	98.311	97.766	97.297
	B	97.279	96.622	97.369	
	M	97.952	96.959	97.727	
ResNet50 with Fuzzy Ranking using modified Gompertz Function	N	98.986	98.986	98.916	98.986
	B	99.321	98.649	99.152	
	M	98.658	99.324	99.298

## Discussion on the findings

As shown in Figs. 7, 8 and 9 and in Table 2, the standalone VGG11, Inception v3, and ResNet50 models performed well in the breast cancer classification problem since the overall accuracy of the models attained in the range from 95.833 to 96.622%. The classification results are further enhanced using the Ensemble approach using Weighted Average, Sugeno Integral, and Fuzzy ranking based on modified Gompertz functions. As compared with the performance of standalone transfer learning models, the classification performance of the ensemble method using the weighted average approach is not substantially improved. The attained range of overall classification accuracy is 96.059 to 96.847% and this is because this approach of using weighted average is a static methodology where the weights of the classification models are not dynamically altered during the final prediction. After replacing the Sugeno integral function in place of the Weighted Average, there is a notable improvement in the attained results, that is, the attained range of overall classification accuracy is 96.396 to 97.297% whereas the values of Precision metric is also increased for each output class and is range from 95.667 to 97.952%, values of Recall ranges from 95.608 to 98.311% for all output classes, and values of F1 score ranges from 96.479 to 97.766% for all output classes.

However, the results need to be improved further for attaining the design of a promising CAD framework. To address this and to improve the results of breast cancer classification, the work proposed the Fuzzy ranking ensemble of TL models using a modified Gompertz function. As discussed in subsection 4.3, the proposed method provides a superior classification performance of 97.072% of overall classification accuracy for VGG11, 98.086% for Inception v3, and 98.986% for the ResNet50 model. This in turn, the precision, recall, and F1 scores are attained best for all classes as 99.321%, 99.324%, and 99.298%. Herein, it is noted that the above superior metric results are attained for the ResNet50 model. Also noted that the Inception model always looks for reduced computation whereas the ResNet model works on enhancing the classification performance with improved depth of the architecture. The above discussions are validated using the static validation kappa $(\kappa)$ values and the attained values are compared against the overall classification accuracy for each employed model and plotted in Fig. 10. As shown in Figure, the superior results attained for the proposed work are validated and so a maximum $\kappa$ value of 95.6 for VGG11, 97.1 for Inception v3, and 98.5 for ResNet50 are attained when fused with Fuzzy Ranking using modified Gompertz function.

For further validation, a one-way analysis of variance (ANOVA) is conducted to compare the overall accuracy metric of the employed transfer learning models. This allows us to verify the substantial differences among the classification models. Since the overall accuracy performance metric is commonly calculated for all three classes, ANOVA is performed against the overall accuracy values. The statistical analysis conducted on the overall accuracy of the classification models is summarized in Table 3. Here, SS, and DF represent the Sum of Squares and Degrees of Freedom. As from this table, the *p*-value is greater than 0.05, so it failed to reject the null hypothesis. Thus, there is no significant difference in overall accuracy scores among the employed classifiers for breast tumor classification. Since the proposed model’s overall accuracy is not significantly different from those of other models (VGG11, Inception v3, ResNet50), the ANOVA scores as given in Table 3 illustrate that the proposed model performs comparably well. Table 4 gives a summary of a comparison of the proposed research with the existing literature study.

**Table 3:** Statistical scores of ANOVA conducted on overall accuracy obtained for the employed classification models

Source of Variation	SS	DF	MS	F	*P*-value	F critical
Between Classification models	2.454219	2	1.227109	1.797117	0.220454	4.256495
Within Classification models	6.145388	9	0.682821	-	-	-
Total	8.599607	11	-	-	-	-

**Table 4:** Performance comparison of the proposed framework with the existing literature

Research Works	Methodology	Overall Accuracy (%)
Wei et al. [9]	Bi-Convolutional Neural Network	97.97
Faisal et al. [7]	Gradient boosting with majority voting	90
Khuriwal et al. [10]	Adaptive voting based Ensemble model	98
Rajaraman et al. [38]	Stack based Ensemble approach	98.07
Naji et al. [5]	Majority voting based Ensemble with machine learning algorithms	98.1
Bhowal et al. [12]	Choquet fuzzy integral based Ensemble using deep learning	95
Proposed Work	ResNet50 with Fuzzy Ranking Ensemble using modified Gompertz function	98.986

## Limitations of the proposed model

In general research problems such as critical breast cancer classification problems, all the researchers are working on providing robust and promising results. However, the classification framework will be struck at any of the steps such as dataset selection, preprocessing, data cleaning, experimentation, and classification. Our proposed CAD framework model equipped with ResNet50 with Fuzzy Ranking using modified Gompertz function provides well discriminative power in classifying benign mammogram inputs but comparatively obtained mere lower performance (Table 2) on discriminating other classes (normal and malignant). This will be taken care in our future extension of this work.

## Conclusion and future work

Being a mortal disease among global women, breast cancer’s incidence rate is progressing gradually year-by-year. For addressing this societal healthcare problem, the need for the design of a robust classification framework is always appreciated among the research community. For such type of mortal cancer, the mammogram images play a vital role in the screening process of the disease and so the work employs the multimodal mammograms from four distinct databases. Accordingly, 986 mammogram images for each output class of normal, benign, and malignant cases have been taken into consideration. These mammogram images are preprocessed for its pectoral muscle and noise removal using global thresholding and adaptive median filter. Transfer learning models are becoming so popular for their efficient and easier implementation. The work utilized the higher representation capability of three transfer learning models such as VGG11, Inception v3, and ResNet50 for a three-class classification problem. The obtained classification results are found to be good but the results are further improved using a fuzzy-ranking-based ensemble approach using the modified Gompertz function. And found that the ResNet50 together with the above ensemble approach provided a superior classification performance of 98.986% of overall accuracy with the validated kappa of 98.5. For comparison, the weighted average and sugeno integral functions were employed with the transfer learning models and the results revealed that the proposed work outperformed all other ensemble approaches for the breast cancer classification problem. The future work extension and recommendations are as follows: the segmentation problem will be taken into consideration using the proposed framework and utilizing clinical mammogram images for testing and evaluation of the proposed work. In addition, the proposed model will be evaluated on different breast imaging modalities such as breast histopathology, breast ultrasound images, and breast thermographic images.

## Data Availability

The data that support the findings of this study are publicly available at https://bcdr.eu/ (Breast Cancer Digital Repository), http://peipa.essex.ac.uk/info/mias.html (MIAS), https://www.academicradiology.org/article/S1076-6332(11)00451-X/fulltext (INbreast), https://complexity.cecs.ucf.edu/cbis-ddsm/ (CBIS-DDSM).

## References

[1] Abirami C, Harikumar R, Chakravarthy SS. 2016. Performance analysis and detection of micro calcification in digital mammograms using wavelet features. In 2016 International Conference on Wireless Communications, Signal Processing and Networking (WiSPNET) (pp. 2327–2331). IEEE.

[2] SR SC, Rajaguru H. 2021. A Systematic Review on Screening, Examining and Classification of Breast Cancer. 2021 Smart Technologies, Communication and Robotics (STCR), pp.1–4.

[3] Sung H, Ferlay J, Siegel RL, Laversanne M, Soerjomataram I, Jemal A, Bray F. Global cancer statistics 2020: GLOBOCAN estimates of incidence and mortality worldwide for 36 cancers in 185 countries. Cancer J Clin. 2021;71(3):209–49. 10.3322/caac.21660 33538338

[4] Sannasi Chakravarthy SR, Rajaguru H. Detection and classification of microcalcification from digital mammograms with firefly algorithm, extreme learning machine and non-linear regression models: a comparison. Int J Imaging Syst Technol. 2020;30(1):126–46. 10.1002/ima.22364

[5] Naji MA, El Filali S, Bouhlal M, Benlahmar EH, Abdelouhahid RA, Debauche O. Breast Cancer prediction and diagnosis through a New Approach based on Majority Voting Ensemble Classifier. Procedia Comput Sci. 2021;191:481–6. 10.1016/j.procs.2021.07.061

[6] Chakravarthy SS, Rajaguru H. Automatic detection and classification of mammograms using improved extreme learning machine with deep learning. IRBM. 2022;43(1):49–61. 10.1016/j.irbm.2020.12.004

[7] Faisal MI, Bashir S, Khan ZS, Khan FH. 2018. An evaluation of machine learning classifiers and ensembles for early stage prediction of lung cancer. In 2018 3rd international conference on emerging trends in engineering, sciences and technology (ICEEST) (pp. 1–4). IEEE.

[8] Mughal B. Early Detection and Classification of Breast Tumor From Mammography (Doctoral dissertation, COMSATS Institute of Information Technology, Islamabad). 2019.

[9] Wei B, Han Z, He X, Yin Y. 2017, April. Deep learning model based breast cancer histopathological image classification. In 2017 IEEE 2nd international conference on cloud computing and big data analysis (ICCCBDA) (pp. 348–353). IEEE.

[10] Khuriwal N, Mishra N. 2018, March. Breast cancer diagnosis using adaptive voting ensemble machine learning algorithm. In 2018 IEEMA engineer infinite conference (eTechNxT) (pp. 1–5). IEEE.

[11] Thuy MBH, Hoang VT. Fusing of deep learning, transfer learning and gan for breast cancer histopathological image classification. In: International Conference on Computer Science, Applied Mathematics and Applications. Cham: Springer; 2019. p. 255–66.

[12] Bhowal P, Sen S, Velasquez JD, Sarkar R. Fuzzy ensemble of deep learning models using Choquet fuzzy integral, coalition game and information theory for breast cancer histology classification. Expert Syst Appl. 2022;190:116167. 10.1016/j.eswa.2021.116167

[13] Khan S, Islam N, Jan Z, Din IU, Rodrigues JJC. A novel deep learning based framework for the detection and classification of breast cancer using transfer learning. Pattern Recognit Lett. 2019;125:1–6. 10.1016/j.patrec.2019.03.022

[14] Muduli D, Dash R, Majhi B. Automated diagnosis of breast cancer using multi-modal datasets: a deep convolution neural network based approach. Biomed Signal Process Control. 2022;71:102825. 10.1016/j.bspc.2021.102825

[15] Mahesh TR, Vinoth Kumar V, Dhilip Kumar V, Geman O, Margalam M, Guduri M. The stratified K-folds cross-validation and class-balancing methods with high-performance ensemble classifiers for breast cancer classification. Healthcare Analytics. 2023. 100247,ISSN 2772–4425. 10.1016/j.health.2023.100247.

[16] Sannasi Chakravarthy SR, Rajaguru H. SKMAT-U-Net architecture for breast mass segmentation. Int J Imaging Syst Technol. 2022;1–9. 10.1002/ima.22781.

[17] Debelee TG, Schwenker F, Ibenthal A, Yohannes D. Survey of deep learning in breast cancer image analysis. Evol Syst. 2020;11(1):143–63. 10.1007/s12530-019-09297-2

[18] Sannasi Chakravarthy SR, Bharanidharan N, Rajaguru H. 2022. Multi-deep CNN based experimentations for early diagnosis of breast cancer. IETE J Res, pp.1–16.

[19] Moura DC, López MAG, Cunha P, Posada NGD, Pollan RR, Ramos I, Loureiro JP, Moreira IC, Araújo BM, Fernandes TC. November. Benchmarking datasets for breast cancer computer-aided diagnosis (CADx). Iberoamerican Congress on Pattern Recognition. Berlin, Heidelberg: Springer; 2013. pp. 326–33.

[20] Suckling JP. 1994. The mammographic image analysis society digital mammogram database. Digital Mammo, pp.375–386.

[21] Moreira IC, Amaral I, Domingues I, Cardoso A, Cardoso MJ, Cardoso JS. Inbreast: toward a full-field digital mammographic database. Acad Radiol. 2012;19(2):236–48. 22078258 10.1016/j.acra.2011.09.014

[22] Heath M, Bowyer K, Kopans D, Kegelmeyer P, Moore R, Chang K, Munishkumaran S. Current status of the digital database for screening mammography. Digital mammography. Dordrecht: Springer; 1998. pp. 457–60.

[23] Sannasi Chakravarthy SC, Rajaguru H. Lung cancer detection using probabilistic neural network with modified crow-search algorithm. Asian Pac J Cancer Prev. 2019;20(7):2159. 31350980 10.31557/APJCP.2019.20.7.2159 PMC6745229

[24] Abd Elaziz M, Heidari AA, Fujita H, Moayedi H. A competitive chain-based Harris Hawks optimizer for global optimization and multi-level image thresholding problems. Appl Soft Comput. 2020;95:106347. 10.1016/j.asoc.2020.106347

[25] Weiss K, Khoshgoftaar TM, Wang D. A survey of transfer learning. J Big data. 2016;3(1):1–40. 10.1186/s40537-016-0043-6

[26] Simonyan K, Zisserman A. 2014. Very deep convolutional networks for large-scale image recognition. arXiv Preprint arXiv:14091556.

[27] Sannasi Chakravarthy SR, Bharanidharan N, Rajaguru H. A systematic review on machine learning algorithms used for forecasting lake-water level fluctuations. Concurrency and Computation: Practice and Experience; 2022. p. e7231.

[28] Theckedath D, Sedamkar RR. Detecting affect states using VGG16, ResNet50 and SE-ResNet50 networks. SN Comput Sci. 2020;1(2):1–7. 10.1007/s42979-020-0114-9

[29] Xia X, Xu C, Nan B. 2017. Inception-v3 for flower classification. In 2017 2nd international conference on image, vision and computing (ICIVC) (pp. 783–787). IEEE.

[30] Dong N, Zhao L, Wu CH, Chang JF. Inception v3 based cervical cell classification combined with artificially extracted features. Appl Soft Comput. 2020;93:106311. 10.1016/j.asoc.2020.106311

[31] Dong WM, Wong FS. Fuzzy weighted averages and implementation of the extension principle. Fuzzy Sets Syst. 1987;21(2):183–99. 10.1016/0165-0114(87)90163-1

[32] Sugeno M. An introductory survey of fuzzy control. Inf Sci. 1985;36(1–2):59–83. 10.1016/0020-0255(85)90026-X

[33] Liao J, Wu S, Du T. The Sugeno integral with respect to α-preinvex functions. Fuzzy Sets Syst. 2020;379:102–14. 10.1016/j.fss.2018.11.008

[34] Iliev AI, Kyurkchiev N, Markov S. On the approximation of the cut and step functions by logistic and Gompertz functions. Biomath. 2015;4(2):ppID-1510101. 10.11145/j.biomath.2015.10.101

[35] Haji SH, Abdulazeez AM. Comparison of optimization techniques based on gradient descent algorithm: a review. PalArch’s J Archaeol Egypt/Egyptology. 2021;18(4):2715–43.

[36] Kundu R, Basak H, Singh PK, Ahmadian A, Ferrara M, Sarkar R. Fuzzy rank-based fusion of CNN models using Gompertz function for screening COVID-19 CT-scans. Sci Rep. 2021;11(1):1–12. 34238992 10.1038/s41598-021-93658-y PMC8266871

[37] Mahesh TR, Vinoth Kumar V, Muthukumaran V, Shashikala HK, Swapna B. Suresh Guluwadi, Performance Analysis of XGBoost Ensemble Methods for Survivability with the Classification of Breast Cancer. J Sensors. 2022;2022:8. 10.1155/2022/4649510. Article ID 4649510. 10.1155/2022/4649510

[38] Rajaraman S, Antani SK. Modality-specific deep learning model ensembles toward improving TB detection in chest radiographs. IEEE Access. 2020;8:27318–26. 32257736 10.1109/ACCESS.2020.2971257 PMC7120763

[39] Mahesh TR, Vinoth Kumar V, Vivek V, et al. Early predictive model for breast cancer classification using blended ensemble learning. Int J Syst Assur Eng Manag. 2022. 10.1007/s13198-022-01696-0.

[40] Sahu B, Panigrahi A, Rout SK. DCNN-SVM: a new approach for lung cancer detection. Recent advances in computer based Systems, processes and applications. CRC; 2020. pp. 97–105.

[41] Sahu B, Mohanty S, Rout S. A hybrid approach for breast cancer classification and diagnosis. EAI Endorsed Trans Scalable Inform Syst. 2019;6(20).

